# The utilization of pineapple hump extracts in complete feeds on the quality of free-board rabbit meat

**DOI:** 10.5455/javar.2024.k776

**Published:** 2024-06-06

**Authors:** Galih Ari Wirawan Siregar, Rini Hardiyanti, Uswatun Hasanah, Kennie Cendekia Desnamrina, Ferdy Saputra, Bram Brahmantiyo

**Affiliations:** 1 Department of Animal Science, Faculty of Agriculture, Universitas Sumatera Utara, Medan, Indonesia; 2 Department of Chemistry, Faculty of Mathematics and Natural Sciences, Universitas Sumatera Utara, Medan, Indonesia; 3 Research Center of Animal Husbandry, Research Organization for Agriculture and Food, National Research Innovation Agency of the Republic of Indonesia, Bogor, Indonesia

**Keywords:** Extract pineapple hump, meat quality, pellets rabbit

## Abstract

**Objective:**

The study aimed to identify the effect of pineapple hump extracts in different doses on increasing the chemical and physical quality of rabbit meat.

**Materials and Methods:**

The research stages were carried out with maintenance for two months using a completely randomized design consisting of four treatments and five replicates. P0 = complete ration without pineapple hump extract; P1 = complete ration with the addition of 0.2% pineapple hump extract; P2 = complete ration with the addition of 0.4% pineapple hump extract; and P3 = complete ration with the addition of 0.6% pineapple hump extract. The variables observed were the chemical quality of meat (pH, moisture, carbohydrate, fat, and protein content in rabbit meat), and the physical quality (water holding capacity (WHC), cooking loss).

**Results:**

The data were analyzed using SAS, with significantly different results subjected to Duncan’s further testing. The addition of 0.4% pineapple hump extract in rabbit complete rations had a very significant effect on the WHC of 71.62%. The addition of 0.6% pineapple hump extract also had a significant effect on the protein content of meat, increasing it by 19.17%.

**Conclusion:**

The effects of pineapple hump extract up to 0.6% in a complete diet of weaned rabbits have a positive effect on the physical and chemical quality of rabbit meat, especially on the protein and water-holding capacity of rabbit meat.

## Introduction

The pineapple hump is a waste material or part of the pineapple that is located in the middle of the fruit, has an elongated shape along the fruit, and tastes slightly sweet. Pineapple pits are common in the neighborhood. As an alternative feed ingredient, pineapple hump contains the enzyme bromelain and has a high nutritional value. The bromelain enzyme is more abundant in the ripe fruit hump than in other parts of the pineapple plant [1].

Feed is the determining factor in meat quality. Improving the quality of feed nutrition can help improve the quality of meat. One method is to add bromelain-enzyme-containing pineapple hump extract to the feed. As a result, it can improve feed protein digestibility, resulting in higher meat quality [2]. The bromelain enzyme obtained from pineapple hump extracts is a proteolytic enzyme with the ability to hydrolyze proteins into simpler compounds and cut peptide bonds from substrates that act as catalysts in cells so as to increase feed protein digestibility [3]. The provision of 0.6% pineapple hump extracts has a significant effect on protein digestibility by producing a protein digestibility coefficient of 82.08% [4]. Broiler feed protein was absorbed as body protein deposition, so the mass of meat protein showed an increased broiler body weight. This condition indicates that the higher the protein digestibility, the higher the meat protein mass. Increased protein digestibility indicates a high substrate in the form of protein to increase meat protein mass [5].

In addition to its chemical composition, the physical quality of meat has a significant impact on meat consumption. Color, cooking loss, and water holding capacity (WHC) are all indicators of physical meat quality. Color significantly affects customer attention; in terms of taste and color, rabbit meat resembles chicken meat. The pH value is critical because it can indicate variances in meat quality; the usual pH value is 5.4–5.8. The amount of water that is still present in the meat is calculated using its water-holding capacity. The low value of protein’s ability to hold water will be impacted by the meat’s high liquid output. The value of meat mass that reduces after heating is known as cooking loss. Meat has a low rate of cooking loss and is of high quality since there is little chance that it will lose any of its nutrients as it cooks. Meat softness is a post-cooking condition of meat quality based on how easily it may be chewed while retaining the qualities of live tissues. Based on the preceding description, researchers are interested in determining the chemical and physical meat quality of weaned rabbits from concentrates with the addition of pineapple hump extracts.

## Materials and Methods

### Ethical approval

This experiment was approved by the Animal Ethics Committee of the Indonesian Agency for Agricultural Research and Development, Ministry of Agriculture (Registration Number: Balitbangtan/Balitnak/Rd/01/2021).

### Experimental animals and samples

**Figure 1.**
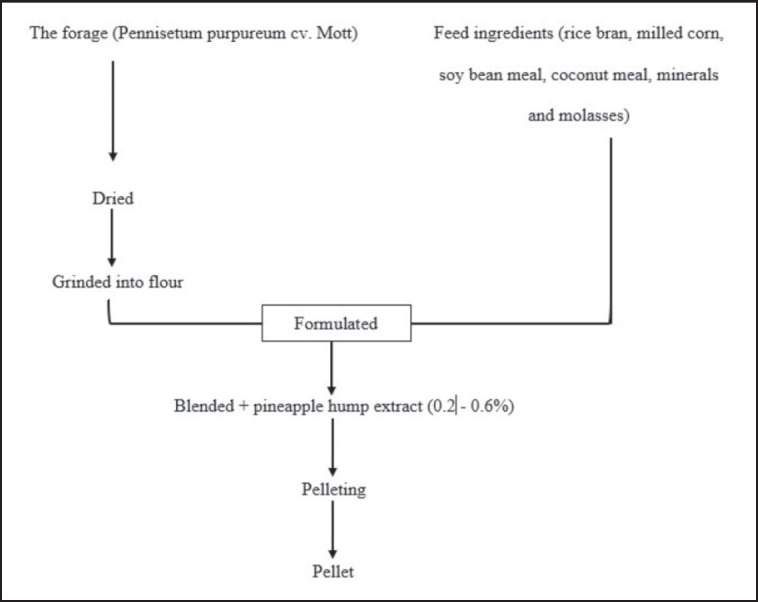
The process of making pellets with pineapple hump extract.

20 two-month-old weaned male rabbits, 20 units of hind leg rabbit meat (16 weeks old) samples that have been treated with the addition of pineapple hump extract as much as 0%–0.6% in the ration. The process of making pellets with pineapple hump extract can be seen in Figure 1.

### Feeding treatment

The feed was modified to meet the demands of weaned male rabbits based on the findings of [6], with protein requirements of 12%–19%, crude fiber of 11%–14%, calcium of 0.9%–1.5%, fat of 2%–4%, and metabolic energy of 2005–2900 kcal/kg. The materials used in this study consisted of complete feed ingredients in the form of forage and concentrates and additional feed in the form of pineapple hump extract, which was formulated into four treatments: P0 (control ratio, without the addition of pineapple hump extract), P1 (ration with the addition of 0.2% pineapple hump extract), P2 (ration with the addition of 0.4% pineapple hump extract), and P3 (ration with the addition of 0.6% pineapple hump extract). The ratio formulation in this study can be seen in Table 1.

### Preparation of roaster rabbit

Rabbits were executed by separating the trachea, jugular vein, carotid artery, and esophagus. After slaughter, the rabbit is suspended by its hind legs, making an incision in the skin between the bones and tendons of the hind joints. The head is separated at the occipito-atlantis joint. The lower front legs and lower hind legs are cut at their elbow joints, and the tail is detached from the base. After the skinning is complete, the entire contents of the abdominal and thoracic cavities are removed, and the individual parts and carcasses are weighed. Carcass parts were then chilled in a refrigerator at 4°C for 24 h [7], after which bones were separated (deboning) to obtain the meat.

### Evaluation of the chemical content of rabbit meat

Observation of the chemical quality of rabbit meat was carried out by sending rabbit meat rabbit samples using the Association of Official Analytical Chemists method [8] to determine the carbohydrate content, measurement of acidity (pH), moisture content, fat content, and protein in treated rabbit meat rabbits.

### Evaluation of the physical quality of rabbit meat

Physical quality testing includes cooking loss according to the method [9] and WHC using the Hamm method. The three components of physical quality are meat color testing using the Android application Color Grab version 3.9.2 and Color Lab version 1.2.

### Experimental design and data analysis

The data were analyzed using a completely randomized design with the formula: *Yij* = µ + τ*i* + ε*ij.*

The mean values were compared with Duncan’s *post hoc* test. Data analysis was conducted using SAS 9.4.

## Results and Discussion

### The chemical quality of rabbit meat

Table 2 contains information regarding the chemical content of rabbit flesh. It can be seen that the protein content of rabbit meat treated with 0.6% pineapple hump extract has the highest protein content (*p *< 0.05) compared to the control and other treatments with a value of 19.17%, the same as research conducted by [10] with a protein content of 19.99%. [11] stated that protein in rabbit meat ranges from 18.2% to 22.1%. The addition of pineapple hump extract with different doses in complete feed produced a meat fat content of 32.6%–16.54% (hindleg). The fat content in this study was higher when compared to [12], which amounted to 20.18% (hindleg), and the study [13] resulted in a lower with a range of 1.27%–1.8% (*Longissimus lumborum (LL)). *The addition of pineapple hump extract did not have a significant effect. The presence of bromelin enzymes in the P3 contributed to its high meat protein level, the more pineapple hump extract that is provided in the feed, the more bromelin enzyme there is, and thus, the greater the protein produced. Based on Table 3, it can be seen that the pH value of weaned rabbit meat with the addition of pineapple hump extract in a ratio ranges from 5.98 to 6.14. The pH value of rabbit meat is not much different from the ultimate pH of meat in general, which is 5.4–5.85. The pH value of rabbit meat in this study is of good quality and suitable for consumption. These results are higher than [14], with a pH range of 5.83–5.98.

### The physical quality of rabbit meat

The WHC of pineapple hump extract in complete diets on the meat quality of weaned rabbits ranged from 68.44% to 71.62%. The highest average was in P2, which was 71.62%, followed by P3, and P0, and the lowest average was in P1, with 68.44%. The WHC in the study produces good quality because there is little water evaporating from the meat, and there is also less water-soluble nutritional content. These results are appropriate and even higher than [15] with a focus on LL, with the average ranging from 68.36% to 70.97%. This is supported by [16], which states that meat with higher WHC has relatively better quality than meat with lower WHC. [17] stated that the standard value of WHC in rabbit meat ranges from 47.99% to 59.21%. The WHC in this study is high because the pH value is also high. Following the statement of [2], which states that the higher the pH value of meat, the value of WHC also increases.

The relationship between treatments can be known by Duncan’s further test, which shows that the WHC treated with 0.4% pineapple hump extract (P2) with an average of 71.62% gives a very significant effect (*p *< 0.01) compared to (P0) feed without pineapple hump extract with an average of 69.69%. This is a result of pineapple hump extract, which contains the protease enzyme bromelain. Peptide bonds in protein content can be hydrolyzed into simpler molecules by this group of enzymes, making high-protein feed easier for the body to digest and increasing the amount of protein in meat.

**Table 1.** Ration formulation.

No	Feed composition	Proportion	Crude protein	Energy metabolism	Crude fiber	Crude fat	Calcium	Phosphor
1	The forage (*Pennisetum purpureum* cv. Mott)	25	2.75	989.25	7.5	0.5675	0.125	0.1
2	Rice bran	15	1.50	247.50	2.25	3	0.006	0.09
3	Milled corn	20	1.72	680	0.586	0.498	0.106	0.106
4	Soy bean meal	25	10.00	560	2,25	1.25	0.05	0.125
5	Coconut meal	12.50	2.3225	275	2	1.5	0.04	0.03625
6	Mineral	0.50	0	0.1685	0	0	0.25	0.125
7	Molasses	2	0.08398	62.22	0.01871	0.0173	0.0164	0.002
	**Total**	**100**	**18.3765**	**2814.14**	**14.6047**	**6.8328**	**0.5934**	**0.58425**

**Table 2.** Chemical content of rabbit meat.

Nutrient composition	P0	P1	P2	P3
Protein	18.32 ± 1.45^a^	15.06 ± 4.92^ab^	11.44 ± 3.59^b^	19.17 ± 2.07^a^
Carbohydrate	3.89 ± 0.43^b^	3.02 ± 0.22^ab^	2.81 ± 0.43^a^	2.66 ± 0.87^a^
Fat	32.61 ± 2.88	36.54 ± 4.54	34.60 ± 4.03	33.87 ± 4.02
Water	70.33 ± 0.32	68.87 ± 0.25	71.93 ± 1.29	70.70 ± 2.03

P0: Current treatment, P1, P2, P3: Rabbit meat contained 0.2%, 0.4%, and 0.6% pineapple hump extract, means in the same line with different superscripts differ significantly (*p* < 0.05).

**Table 3.** Physical quality of rabbit meat.

Parameters	P0	P1	P2	P3
pH	5.98 ± 0.14	6.14 ± 0.17	6.06 ± 0.07	6.09 ± 0.12
WHC (%)	69.69^BC^ ± 0.09	68.44^C^ ± 0.19	71.62^A^ ± 1.01	70.29^B^ ± 1.57
Cooking loss (%)	19.36 ± 0.14	19.38 ± 0.02	19.36 ± 0.03	19.38 ± 0.04

P0: Current treatment, P1, P2, P3: Rabbit meat contained 0.2%, 0.4%, and 0.6% pineapple hump extract, which means in the same line with different superscripts differ significantly (*p* < 0.01).

The cooking loss of pineapple hump extract in complete diets on the meat quality of weaned rabbits is in the range of 19.36–19.38. The outcome of this analysis of variance in this study had no significant effect, but the average in this study was still normal at 19.37. This result is lower than [18], who state that male Bali beef’s average cooking loss varied from 29.16% to 33.91% when fermented pineapple peel was added to the diet. The meat cooking loss in this study fell within a normal range. Meat cooking loss ranges from 15% to 40%, with a typical value of 1.5% to 54.5%. The average cooking loss value of male Bali and the cooking loss value in this study are better than [19], which has rabbit meat cooking loss ranging from 21.50% to 24.00% and a pH value ranging from 5.6 to 5.7, and [20], which has rabbit meat cooking loss ranging from 18.67% to 25.67% with a pH value ranging from 5.58 to 5.80. Low-cooking-loss meat has a relatively good quality compared to large-cooking-loss meat because the risk of losing nutrients during cooking will be less. The greater the cooking loss, the lower the nutritional value of the meat. On the other hand, the lower the cooking loss value, the better the nutritional value because there is less loss of nutritional value.

### The color quality of rabbit meat

The results of the research on the addition of pineapple hump extract to the color with brightness color intensity, yellow color intensity, and red color intensity of the meat of rabbits released from weaning showed that the treatment did not significantly affect it. This is due to the pH value, which is not much different, which ranges between pH 5.98 and pH 6.14 (Table 4), and also due to the same amount of ration nutrients in each treatment and other possibilities due to the same species, sex, and age. Lawrie [2] states that a number of variables, such as nutrition, species, breed, age, sex, stress (amount of activity and kind of muscle), pH, and oxygen, can affect the color of meat.

The color intensity value of the meat brightness in weaned rabbits of pineapple hump extract in the complete ratio of diets ranges from 74.08 to 80.02, with an average of 77.28. The rate at which the bromelain enzyme works to change the color of the meat from red to white varies depending on its concentration. According to [21], the paler or white color of the meat can be caused by the amount of light reflected on the surface of the meat or after the transmission of the meat. The color intensity value of the meat L* and a* in weaned rabbits is higher than [22] with a ratio ranging from 51.8–52.3 and 3.38–3.55, but the b* value is lower with a ratio ranging from 9.44–9.78.

**Table 4.** Color quality of rabbit meat.

Meat colour	P0	P1	P2	P3
Bright	74.08 ± 9.34	80.02 ± 6.33	77.77 ± 6.14	77.25 ± 4.51
Red	12.03 ± 5.83	13.33 ± 4.26	16.65 ± 4.94	15.10 ± 5.67
Yellow	7.13 ± 2.79	7.28 ± 1.70	9.35 ± 2.60	5.96 ± 2.20

P0: Current treatment, P1, P2, P3: Rabbit meat contained 0.2%, 0.4%, and 0.6% pineapple hump extract.

With pineapple hump extract added to a complete feed, the meat of rabbits discharged from weaning ranges in intensity of red color from 12.03 to 16.65, with an average of 14.28. The analysis of variance revealed that the treatment had no significant effect on the reddish color of the meat. This is because rabbits are livestock that are classified as having white meat, and the feed given does not contain myoglobin or other content that causes the red color in animal muscles. The results obtained were not significantly different, and the average in each treatment (P1, P2, P3) was higher than the average P0 (control). This is due to the feed given, which is supplemented with pineapple hump extract, which contains the bromelain enzyme, which functions to break down proteins into amino acid molecules, so that the body digests proteins more easily, which can increase the formation of protein in high-quality meat. Zulfahmi et al. [23] described how meat pigments, which are made up of two different types of proteins called hemoglobin and myoglobin, affect the color of meat.

The content of myoglobin in meat causes differences in the red or white color of the meat. The meat color improves. Ladamay and Yuwono [24] states that the color level is expressed with a value of about −100 to +100. Positive values (+) indicate red color intensity, while negative values (−) indicate green color intensity. This study has an average reddish color of 14.28 in contrast to [21] on the physical quality of rabbit meat which provides an additional feed of soursop leaf flour, and zeolite has an average reddish color of 12.44.

The results of the analysis of variance showed that the treatment had no significant effect on the color (b*) of yellowness. This is because the amount of feed content given is the same, so the rubberonoid content or the content that produces the yellow color contained in the feed is the same, and the addition of pineapple hump extract added to the feed is too little so that the color b* (yellowness) of the meat does not have a significant effect. Ramdani et al. [25] states that pineapple contains carotenoid, xanthophyll, and flavonoid pigments that give pineapple fruit a yellow color.

## Conclusion

Effects of pineapple hump extract up to 0.6% in a complete diet of weaned rabbits had a positive effect on the physical and chemical quality of rabbit meat, especially on protein, WHC of rabbit meat, and fat content from the hindleg, which were higher than LL.

## List of Abbreviations

LL, Longissimus lumborum; WHC, water holding capacity; SAS, Statistical Analysis System; CRD, completely randomized design; AOAC, Association of Official Agricultural Chemists.
